# Conclusions. Seven Lessons to Build an Education Renaissance After the Pandemic

**DOI:** 10.1007/978-3-030-57039-2_8

**Published:** 2020-11-05

**Authors:** Fernando M. Reimers

**Affiliations:** Harvard Graduate School of Education, Harvard University, Cambridge, MA USA; Harvard Graduate School of Education, Cambridge, MA USA

## Abstract

This chapter draws out seven lessons from the cross-country analysis of the six reforms studied in this chapter. These are:

Lesson 1. The power of complex mindsets about education reform.

The six reforms all reflect reliance on the worldviews presented in the five frames of reform: cultural, psychological, professional, institutional and political. Those that have been sustained relied on insights from more of these five frames than those that were short lived.

Lesson 2. Implementation matters considerably.

The chapter discusses how the implementation process in effect recreates a reform, and how the development of an operational strategy defining the details of reform is what in the end most matters to the success of reform. The chapter discusses how the six reforms produced rather distinct operational strategies of seemingly similar components of the reform such as the learning goals for students or teacher professional development. Implementation strategies are also based on implicit theories of how organizations work, and the chapter explains the usefulness of a developmental theory of how organizations evolve to designing strategies that are aligned with the functionings that are possible in a given developmental stage, while also helping the organization evolve towards higher levels of functioning.

Lesson 3. The need for operational clarity.

People can’t execute what they don’t understand, and a reform must be able to translate goals into clear objectives and reform components into clear tasks which can be widely communicated and understood, as well as tracked to discern improvement and course correct when necessary.

Lesson 4. Large scale reform is a journey: Coherence, Completeness and the Five Frames.

The chapter explains how using the five dimensional theory of educational change can support coherence and completeness in a reform.

Lesson 5. Sequencing, pacing and the importance of first steps.

An operational strategy needs to be sequenced attending to ambition of goals, to existing levels of capacity and to institutional stage of development of the system. The first steps in the sequence are consequential because they shape the narrative of reform in ways that have long lasting consequences.

Lesson 6. Staying the course.

Long policy cycles are essential for reforms to be implemented and to produce results, and those cannot be taken for granted. Coherence, communication and participation can garner support that sustains a reform over time.

Lesson 7. Learning from experience to build system level capacity.

Most important to the coherent implementation of a reform is to create opportunities for key stakeholders, at various levels of the system, to learn together as a result of implementing components of the reform. Creating feedback loops and processes for making sense of such information is critical to support such learning.

## Introduction

When it is finally over, we will in all likelihood conclude that the COVID-19 Pandemic was, in many ways, a veritable global calamity. Millions of people were infected, hundreds of thousands died, millions of jobs were lost, economies slowed down, the institutional and financial capacity of governments was burdened crowding out the State’s and private institutions ability to deliver on most fronts. In education, the Pandemic disrupted the normal functioning of education systems, creating challenges for students, families, teachers and systems. The inability to overcome some of these challenges created significant learning losses for many students, amplifying learning gaps within and among nations.

We can anticipate that the long term outcomes of those losses, for individuals and communities, will be dire. Some students will never return to school, others will finish school with less skills than they would have otherwise. These results of the inability to sustain educational opportunity during the Pandemic will influence the future employment prospects of many students, negatively impacting their communities and nations.

But the interruption in the regular functioning of schooling created also a new awareness about the importance of education, greater curiosity and interest about what it was that students were and should be learning in school, it generated more views and even contention about how best to educate students. Perhaps there will be, alongside the obvious education losses, also an educational dividend of the Pandemic, if this interest in making education more relevant and more effective carries on into the Post-Pandemic world.

If this renewed urgency about helping students develop the breath of skills they need to thrive in a world that is volatile and uncertain does indeed continue, we would do well to learn from other system level reforms that have attempted to do just that. The six reforms studied in this book offer seven valuable lessons about how to enhance the capacity of education institutions to pursue more ambitious education goals. These lessons focus on the mindsets about reform, the importance of the implementation process, the need for operational clarity, the journey of reform, sequencing and the role of first steps, staying the course and learning from experience. I expand on each of those seven themes below.

## Lesson 1. The Importance of Mindsets About Education Reform

The six reforms examined in this book demonstrate that large scale system reform was already happening throughout the world preceding the Pandemic, as governments transformed education systems to pursue more ambitious goals. The approaches followed by these reforms can be usefully characterized using the five frames I have argued explain how reformers view the process of educational change: cultural, psychological, professional, institutional and political (Reimers, 2020). While each of these six reforms relies on more than a single frame, countries can be divided in two groups in terms of how they appear to have drawn on the worldviews that characterize these five frames.

Whereas in Ontario, Singapore and Zimbabwe there are clearly recognizable elements of all five frames, in Mexico, Pakistan and Kenya, only one or two frames are clearly dominant. While the three reforms that reflect a more comprehensive view of the process of change vary, with reforms in Ontario and Singapore having been in place much longer than Zimbabwe’s, it is noticeable that Zimbabwe’s reform appears to have attempted, and to some extent accomplished, ambitious change in an obviously tumultuous period characterized by considerable political and economic turmoil. In contrast, the reforms in Pakistan, Mexico and Kenya, reflecting a more limited set of worldviews in their implementation, appear to have faced more challenges, in particular regarding their ability to achieve the intended changes and in terms of sustainability.

Ontario’s reform reflects a political and professional perspective, focusing on supporting the professional autonomy and development of teacher capacity, strengthened by political support from teachers and teacher unions. Significant reliance on creating structures and processes to shape the reform and to develop the capacity of teachers indicates strong use of an institutional perspective. In particular, the use of data and frequent feedback loops to guide instructional improvement and management decisions, and the attention to fostering coherence across the three levels of the system (the school, the middle and the system) reveal a distinct reliance on and institutional view of how to help various stakeholders develop a shared understanding of ‘the system’. The reform leaders were clearly attuned to expectations of teachers and of the population to replace the conflicted nature of relations between government and school for more collegial relationships, and they relied heavily on consultative and participatory approaches early on, suggesting an awareness of elements of a cultural perspective. While the reform relied on ideas about improvement that had a base on academic knowledge generated by research, and several of the processes created had the purpose of strengthening the role of evidence based knowledge to guide the reform, a psychological perspective, relying on science based knowledge to guide the reform, was conspicuously absent as a strong professional perspective prioritized knowledge based on practice and generated by practitioners, over knowledge based on academic research.

Singapore’s reform of teacher education reflects also a blend of psychological, professional and institutional worldviews. In aligning the preparation of teachers with an ambitious curricular vision this reform drew on learning sciences to design deep learning experiences for teachers, and created institutional changes to sustain those reforms over time. There is no apparent reliance on a cultural frame, other than in the fact that the reform continued and deepened a ‘culture of education’ which had already established the importance of twenty-first century skills as an aspirational goal to drive reform efforts. The deft use of political worldviews is evident in the absence of political contention regarding the reform, as the reform benefited from a solid understanding of how change is accomplished in Singapore’s education system, with an incremental approach, building on and honoring previous efforts, and with quiet, but palpable, support from the higher levels of education leadership in the Ministry of Education and in the National Institute of Education, and perhaps also in the Cabinet.

Mexico’s reform is first and foremost an institutional and political reform, incorporating only in subsequent stages components reflecting a psychological and a professional perspective. There is no evident use of a cultural frame in the design of an unapologetic top down reform, although arguably the reform built on a widespread perception that the education system was not adequately serving the needs of students. While consultative processes were deployed once the reform began to formulate the curriculum component, 3 years into the reform, and the design for the reform included structures and processes designed to cultivate local participation and support, very much along the lines of Ontario’s, those components were only announced once a public narrative opposing the reform had crystalized. The opposing unions and other detractors of the reform created a narrative about the reform as a punitive, anti-labor effort, from which it never recovered. The contrast between Mexico’s reform and Zimbabwe’s is interesting, as both aimed at significant elevation of curricular goals. Even though Zimbabwe’s reform was launched in a much more embattled political and economic context, it rapidly sought to advance a multiplicity of components at once, whereas Mexico, in a much more favorable political and economic situation, advanced the institutional reforms rapidly, but was more gradual, arguably slow, in bringing about the curricular and professional changes, in effect running out of time and of political capital to effectively achieve those. While counterintuitive, Zimbabwe’s bold move to attempt the impossible, to do much without apparent political, financial or institutional capital to achieve it, may have worked better than the more conservative, gradual, approach followed by Mexico.

Punjab’s reform is principally an institutional reform, benefiting from skillful and consistent political support over an extended period. Through institutional mechanisms, the reform also tried to professionalized teaching, for instance changing the rules for teacher appointments to be more meritocratic, increasing the number of teachers in schools so that teachers could spend more time preparing their lessons, increasing the number of school coaches so that they could devote more time working with teachers on improvement strategies. There is no evidence that a cultural perspective informed what was unquestionably a top down reform with strong political support from the Prime Minister, as had been the case with the British reform on which the Punjab’s reform was inspired. The absence of insights from learning sciences that characterize a psychological perspective is conspicuous in Punjab’s case.

Kenya’s reform draws largely on worldviews reflecting cultural and institutional frames, comprising a curriculum reform that requires responding to and supporting changes in broadly held expectations about schooling and its ways, and changes to structures to support the new curriculum. There is no obvious reliance on a political perspective, other than in the initiation of the reform via a pilot, which allowed to test not only the components of the reform, but the readiness of key stakeholders to embrace it. A psychological perspective is absent, except in the definition of some of the competencies which are included in the new curriculum. For example, the reliance on a train the trainer model, with very limited extended support in school to teachers, to build the capacities of the ambitious goals and pedagogies that are the core of the reform, ignores much of the research-based knowledge on effective teacher education. A professional perspective is also lacking in the reform, in glaring contrast to the reforms in Ontario and Singapore in that respect.

Zimbabwe is an institutional reform focusing on transforming a content based into a competency based curriculum. But the reform, at least in the early stages in which it is in 2020, relies also on worldviews that reflect cultural, psychological, institutional and political perspectives, even if somewhat superficially. The ambition of the reform, and the rapid pace at which it is being implemented, is counterintuitive given the high political and economic volatility in which it is taking place. It is possible that the reform is benefiting from the dividend of what was for decades a relatively highly professionalized education system, until successive economic crises caused many of the teachers to abandon the system. Reflecting reliance on a cultural frame, the reform consulted extensively with parents throughout the design and implementation process. The psychological perspective is reflected in the competencies included in the curriculum and in the thoughtful design of the curricular sequences, as well as in the multiplicity of approaches and structures envisioned to support teacher professional development. It also recognized the need to develop the professional skills of teachers to teach the new curriculum, reflecting a professional perspective. An institutional perspective is visible in the deployment of social and institutional structures to support teacher and learning and monitor quality of delivery. Finally, the reform relied on top down political support for implementation, a mixed blessing in a context of high political volatility.

## Lesson 2. Implementation Matters Considerably

Reforming education systems is as much about, if not more, implementing educational change than about designing policy reform. What is a reform that is not implemented, or that is implemented in a way that distorts the intended objectives?

There are at least two reasons the topic of implementation merits careful consideration when transforming education systems at scale in order to help students gain a broader range of competencies. The first is, to put it bluntly, that a reform is not more than what is implemented. Reforms that remain on policy documents have little use other than adorning government bookshelves or providing fodder for government rhetoric. In a world of almost instantaneous communication and increasing transparency, however, it is not easy to trick the public into believing in a reform which exists only in the imagination of its leaders, or on paper, at least not for too long. The process of implementation often ‘translates’ policy initiatives into rather different operational activities than those intended and that translation in effect ‘recreates’ reform. This was the powerful insight developed by Michael Lipsky in his seminal book ‘Street-Level Bureaucracy’ in which he explained how policy is sometimes implemented in unexpected and unintended ways as Street-level bureaucrats ‘make policy’ because they exercise discretion resulting from their professionalism and relative freedom from oversight and authority performing tasks which cannot be scripted (Lipsky, 1980). Anticipating what Frederic Laloux in his book ‘Reinventing Organizations’ describes as a ‘Pluralistic’ view of organizations, which will be explained later in this chapter, Lipsky explains that the transformation of policy may also result from Street-level bureaucrats disagreeing with the views of their managers.

As implementation defines the important details of policy, it is those details that shape the fate of reform. Fullan and Gallagher refer to the importance of developing a ‘nuanced’ understanding of those details of reforms:

Two systems can each produce all of the right foundational documents (annual improvement plans, SMART goals, literacy strategies, even deep learning strategies). They can believe in and provide professional development for teachers and express trust in their school staff, but one gets results and sustainable change and the other, actually most, do not. In our view, it is in the details that the difference is made. It is found in details such as the ways in which trust is built, the degree to which the implementation is nimble and flexes as it needs to in response to what is happening in the situation. (Fullan & Gallagher, 2020, viii).

Sometimes the details that matter are not as nuanced as Fullan and Gallagher describe, but are reflected in clear and visible ways in how various terms that define the components of a reform are operationally defined. The reforms studied in this book show that the same terms are commonly used to refer to components that, in practice, are rather different. For example, all of these reforms included a component to ‘build teacher capacity’ in one way or another, but this component was translated into very different activities in each case. In Ontario, for example, the component to build teacher capacity included a saturation of structures and processes to increase the knowledge and skills, and more importantly the shared understanding, of a very large number of stakeholders, from teachers to staff in the provincial Ministry. Capacity building involved creating a specific unit in the Ministry (the Literacy and Numeracy Secretariat) where about forty specialists were tasked to design improvement strategies to build teacher and leadership capacity focused on improving student outcomes in literacy and numeracy. In addition, each school created a team tasked with developing and implementing a student success initiative. A specific network was established to mobilize knowledge and support of school-based improvement strategies, partnering the directors of the five highest achieving districts with those from the eighteen lowest achieving districts to support the development of literacy and numeracy improvement strategies.

In contrast to Ontario, which focused on developing teacher professionalism and building teacher capacity primarily in their schools focused on their literacy and numeracy instruction, the focus of professional development in Singapore was much broader than two subject domains. Focusing on teacher initial preparation, Singapore’s reform of teacher initial preparation was aligned to a twenty-first century conception designed to help develop teachers holistically and included developing clear competency frameworks for teacher graduates, that included themes such as **V**alues of learner-centeredness, teacher identity, and service to the profession and community, **S**kills required by an educator, and in-depth subject-matter **K**nowledge, and that focused on three domains: professional practice, leadership and management, and personal effectiveness. The aims of Singapore’s reform included helping teachers develop their own teaching philosophies, developing the habit of reflection on their practice supported by an e-portfolio, providing teacher candidates opportunities to learn from experiential learning and other deep learning pedagogies.

Contrasting to Ontario and to Singapore, the Mexican reform also included components to build teacher capacity but the specific operational mechanisms were developed in much more general terms than in Ontario or Singapore, were announced much later in the implementation cycle, towards the end of the term of the administration, and received limited funding and support from the States, with the ensuing limitations to implementation. Whereas in Ontario and Singapore the reforms began with professional development, this component in Mexico was an afterthought to the institutional and political reforms.

The Punjab reform, which also aimed at strengthening teacher capacity, did this in ways that are, by comparison to Ontario and Singapore, significantly more superficial, lacking the intensity of efforts that was apparent in these two reforms. In Punjab, professional development involved developing teacher training self-instructional modules, providing training to use them, and hiring teacher coaches to work with teachers. This ‘dosage’ of opportunities to develop new skills, contrasts to the rich set of structures, processes and opportunities that Ontario established to strengthen teacher capacity or to the deep changes that Singapore introduced in teacher preparation to help teachers develop twenty-first century competencies, and is more similar to what was done in Kenya through a train the trainer approach and short courses, which the pilot of the reform showed was insufficient to develop the capacity to teach an ambitious curriculum.

Kenya’s reform, which attempted more ambitious curriculum change than the Punjab’s or Ontario’s, also relied on building teacher capacity. A pilot of the curriculum reform shows how easy it is to underestimate the needs for professional development to implement a demanding reform. The full implementation of the reform was postponed once the pilot revealed that the professional development which had been planned, consisting of 1–3 week courses to familiarize teachers with the new curriculum and the associated pedagogies, with some reinforcement during breaks, delivered via a train the trainer model, was deemed insufficient by the teachers participating in the pilot to equip them with the skills to teach the new curriculum. The contrast of this train the trainer model implemented in short sessions with the approach followed in Ontario could not be greater, even though both reforms included teacher professional development as a crucial reform component.

Zimbabwe’s reform, similar to Kenya’s in its focus in replacing a content-based curriculum with a competency based curriculum, followed in turn a different approach to professional development, also relying on a train the trainer model, but extending into supporting teachers in schools, more similar to the approach followed in Punjab, and what was planned in Mexico, but more shallow than what was done in Ontario.

Just as the operational translation of the concept of building teacher capacity in these six reforms reveals consequential differences, similar important differences in the details of implementation of these reforms are found with respect of other components. Not least among them the operational definition of the student learning outcomes that these reforms attempted to influence. All of the reforms studied in this book were predicated in preparing students for the increasing demands of the twenty-first century, with two of them, Ontario and Punjab, translating this aspiration on a sharp focus on literacy and numeracy, and also access to school in the case of Punjab and high school graduation in the case of Ontario, and four of them focusing on a wider set of learning goals for students. Those aspirations, however, were translated into different operational descriptions of what particular competencies students should be developing. In Mexico, the new education model outlined a detailed, ambitious and comprehensive taxonomy of competencies which guided the curriculum, arguably the most comprehensive and clearly developed of the six reforms studied in this book, and the one most aligned with the breadth of competencies discussed in Chap. 10.1007/978-3-030-57039-2_1. In Singapore, similar ambitions were articulated not for students but for teachers, in order to help them lead twenty-first century instruction. None of the other reforms examined in this book had similarly clear and ambitious expectations for what teachers should learn in order to lead twenty-first century education.

In Ontario, in contrast, the learning goals were initially narrowly focused on literacy and numeracy, and even as those goals expanded partway through the reform, the expansion was still largely about broader ways to understand literacy and numeracy as foundations to a broader set of competencies. In Pakistan, education quality was also translated into an operational focus on literacy and numeracy.

In Kenya the ambition to prepare students for the twenty-first century translated into a competency-based curriculum, a goal to help all students begin secondary school, eliminating exams, and creating a variety of pathways at the secondary level with an emphasis on technical and vocational tracks. Kenya’s curriculum focused on seven core competencies: communication and collaboration, critical thinking and problem-solving, creativity and imagination, citizenship, digital literacy, learning to learn, and self-efficacy, all of them broader than the learning goals in Ontario or Punjab, more in line with those in Singapore, considerably less detailed than those in Mexico.

In Zimbabwe, the shift from a content based to a competency based curriculum emphasized higher order thinking skills, the development of national identity, preparing learners for life and work, for life-long learning, for participatory citizenship, peace and sustainable development, and for participation, leadership and service. These goals too were broader than those pursued in Ontario or Pakistan, and very similar to those pursued in Kenya.

This variability in the operationalization of education policy goals suggests that we need a language of implementation. Much of the language we have is more adequate for policy reform, than it is to guide the implementation process. It is high level conceptual language, useful for logical analysis, but insufficient to guide the operational planning of reform. The robust literature on the ‘drivers’ of policy change, the ‘policy levers’ that may lead to higher student outcomes, yields a working consensus on what policies matter, even as that knowledge may be limited by the limited range of countries where the research from which those drivers were identified was conducted as discussed in the first chapter of this book. But that literature is thin when it comes to defining what those terms mean for implementation, as a result the most critical details to inform the design of implementation strategies are left to the discretion of administrators and other Street-level bureaucrats. It is one thing to say that reforms should have clear goals for students, or even that those goals should equip them with twenty first century skills in cognitive, interpersonal and intrapersonal domains, it is another thing altogether to translate those aspirations into specific standards and curriculum, with the level of operational details that would allow two different people to have shared clarity as to what those goals mean in practice.

We often refer to the combination of policy levers, to the mix of components that are included in a reform, and to the assumptions we make about how the actions that a reform will support will produce particular results, as a theory of action. A theory of action can be expressed with a methodology called the logical framework approach, which makes explicit the underlying hypotheses of a policy or a program about how particular inputs will support activities which will in turn lead to immediate results and eventually to long term outcomes. A theory of action is a statement that makes visible how a policy is supposed to achieve its intended results. It’s implementation ‘in theory’.

For example, the National Literacy and Numeracy Strategy advanced in the United Kingdom by Tony Blair, which served as the inspiration for the reforms in Ontario and the Punjab, comprised six key foundations: define clear and ambitious standards, use data to monitor performance towards targets, devolve responsibility to teachers and schools, access best practice and high quality professional development, create accountability, and focus on low performing schools. These are all elements that can be integrated in formulations of the sort ‘if I do A then B will happen’. For example, ‘If standards are clear and ambitious and teachers are given the freedom to design how to improve, and they are provided the support from peers and access to networks of colleagues, they will discover more effective ways to teach, will enact changes to their pedagogies and as a result students literacy skills will increase’.

In Ontario, for instance, the basic theory of action of the reform was that increasing teacher professionalism and accountability would lead to improvements in literacy, numeracy and high school graduation rates. The key hypotheses of the reform could be phrased as: ‘if educators are provided the autonomy, and the professional development and support, to design their own plans for improvement, their pedagogical efficacy in literacy and numeracy will increase, leading to gains in student learning outcomes in those domains and greater personalization in high schools will lead to greater student engagement and to increases in graduation rates.’

In Singapore, the basic theory of action was that in order for teachers to help their students develop twenty-first century skills, they themselves must display them, and in order for them to be able to lead deeper learning processes, they must have experienced such teaching and learning. Teacher education was aligned to an explicit and ambitious framework of teacher competencies, and a number of changes to the preparation programs were introduced to specifically develop those competencies.

In Mexico, the theory of action was that greater teacher professionalism and an ambitious curriculum would support students to develop twenty-first century skills. To achieve greater professionalism the reform changed the rules governing access to and promotion to the teaching career, designed a new educational model, which specified not only a curriculum, but more autonomy and local participation for schools. An implicit theory of action driving the reform was that the control of the teacher union over the careers of teachers had to be eliminated and replaced by public rules and processes that recognized professional effort, skills and efficacy in order to develop the education system towards greater professionalism.

In Pakistan, the basic underlying theory of the reform was similar to that of the British reform on which it was inspired, although the goals included access to school, which was not a goal in the United Kingdom. Both reforms aimed at improving quality in public schools and at strengthening the capacity for monitoring and evaluation to improve accountability at all levels of the system. The reform was based on strengthening management capacity, largely creating systematic reporting of information on the status of implementation of reform activities to various management levels in the system. While there was also a component to strengthen teacher quality, through new norms for teacher recruitment, increase in the number of teachers, in-service professional development through self-instructional modules and with support from teacher coaches, these activities were in the shadows of the much more salient emphasis on using information systems to strengthen management capacity.

In Kenya and Zimbabwe the basic reform theory was that a competency based curriculum, supplemented by teacher professional development, would lead to changes in the core of instruction which would in turn help students gain the competencies necessary to meet the demands of the twenty-first century. The necessary support for teachers to be able to teach the new curriculum seems to have been underestimated in both cases. In Kenya, a pilot implementation concluded that the 1–3 week training on the new curriculum was insufficient. In Zimbabwe financial constrains limited the implementation strategy.

Given the variability in the operational definitions of the core policy components of system level reform included in the theories of action of system level reforms, without an explicit guide to translate the theories of action of reform into operational strategies, the operationalization will be left to administrators and teachers who will make consequential decisions about what it is that will actually be implemented. The fate of the reform is entirely contingent on those choices of street level bureaucrats, taking place with much less scrutiny than the public debate that accompanies the identification of policy alternatives. One valuable role for using a Logical Framework is that it makes such translation transparent and open to public scrutiny. This is critical for project management and for evaluation. As we take stock of how reforms have been implemented we complement the existing knowledge about ‘policy reform in theory’ with essential knowledge about ‘policy reform in practice’. To build that knowledge base we need greater focus on the implementation of reforms, examining the operational translations of policy ideas, not about policy ideas themselves, as the various chapters in this book do.

A second reason more attention to implementation is necessary is because reforms depend on unexamined mental models that relate policy change to implementation as much as they depend on the particular components and mix of actions that form the policy.

A theory of action is not just about the particular components of the policy and how they are expected to work in practice. While most typically implicit and invisible, there is a second order set of assumptions undergirding a theory of action. These are assumptions about of how education organizations work, and they form mental models, much like the mental models I represented in the five frames about reform discussed earlier. They are often based on a theory of mind about what explains the behavior of the people who make an education system work.

A common mental model is: ‘If a policy mandates a change, people will comply’. The model assumes that through instruments of incentives and coercion, the education authority can influence and predict the behavior of implementors. An example of that model in practice would be the assumption that ‘If the education authority provides each classroom with classroom libraries, and directs teachers to provide students the freedom to pick books of their choosing and to read them, teachers will comply, students will pick the books, and read them’. This mental model, that people will do as told by those in positions of formal authority, is widespread and often unexamined. It assumes compliance and assumes that particular roles provide individuals in position of authority the power to restrict or encourage behavior in others. This is clearly a top-down mental model that sees an educational institution as a command and control hierarchy. Lipsky’s book ‘Street Level Bureaucrats’ challenged the validity of this model suggesting that implementors could subvert a policy rather than comply. A significant contribution of Lipsky’s book is in helping those in organizations, policy makers as well as implementors, recognize that different people may hold different mental models about how organizations work. This understanding is crucial, it is a powerful idea to help develop a more complex theory of mind to the assumption that people are cogs in a machine, so that those in the organization can communicate and collaborate.

It is certainly the case that some education organizations work as command and control hierarchies, and that many see them as ‘machines’ where people are ‘cogs’, but it is also the case that not all of them do, and that they may not be the most adaptive form of functioning to address certain education challenges. Furthermore, people working in organizations that operate as command and control hierarchies may differ in how they view the value of operating in that way for the task at hand. In education organizations the ‘cogs’ have a mind about whether they and others are indeed ‘cogs’ in a machine or instead people with agency and choice.

There are clear advantages to command and control hierarchies. They provide predictability, allow the organization of human effort in service of large goals, whether those include organizing agricultural societies, building cathedrals or pyramids, engaging in battle, manufacturing cars, or rapidly expanding access to school to enroll most children, as was done in the decades following the inclusion of education as a human right in the Universal Declaration of Human Rights. From the vantage point of this mode of organization enhancing implementation is about enhancing the institutional capacity of the implementation machine to execute policy, as implementors learn to do as told. Activities such as building the technical skills of people at various levels of the system or using information more effectively can enhance capacity, namely ‘the capacity to do as instructed’. The Punjab reforms reflect this organizational mental model.

But command and control hierarchies have their limitations too, especially when the challenge is not to achieve predictable results in a stable world, but to respond to major changes in context – as in a Pandemic, where teachers who are close to students and families may have more knowledge about the conditions the children are in, or their needs, than education authorities – or to invent new ideas or products – as in reforming education to make it more relevant to a world that is volatile and uncertain.

Frederic Laloux offers a valuable conceptualization of the evolution of organizations over the course of human history (Laloux, 2014). In Laloux’s account of the historical development of organizations, they have evolved trough the following seven stages:

Reactive, the earliest developmental stage taking place between 100,000 to 50,000 BC. These organizations were bands of a few people who associated for survival.

Magic, these were tribes forming about 15,000 years ago of up to a few hundred people organized mostly for survival and to handle the demands of the present.

Impulsive, forming about 10,000 years ago and comprising chiefdoms and protoempires. The major breakthrough in these early organizations was division of labor and role differentiation.

Conformist, starting around 4000 BC in Mesopotamia these represent a shift from chiefdoms and survival horticultural societies, to the organization of agriculture, states and civilizations, institutions, bureaucracy and organized religion. These are the first organizational forms that can achieve long term goals, shaping the future.

Achievement, the product of the Renaissance and of the Enlightenment, these complex organizations enabled significant material progress and much liberation and advancement to individuals. This stage “moved us away from the idea that authority has the right answer (instead it relies on expert advice to give insight into the complex mechanics of the world) and brings a healthy dose of skepticism regarding revealed truth. It has allowed us to engage for the first time, in the pursuit of truth regardless of religious dogma and political authority, without having to risk our lives. We have become capable of questioning and stepping out of the conditions we were born in, we are able of breaking free from the thoughts and behaviors that our gender and our social class would have imposed upon us in earlier times.” (Laloux, 2014, p. 25).

Pluralistic, a form of organization that acknowledges that all perspectives deserve equal respect, not only ‘what works’. “It seeks fairness, equality, harmony, community, cooperation and consensus” (Ibid p. 30). “For people operating from this perspective, relationships are valued above outcomes. For instance, where Achievement-Orange seeks to make decisions top-down, based on objective facts, expert input, and simulations, Pluralistic-Green strives for bottom-up processes, gathering input from all and trying to bring opposing points of view to eventual consensus” (Idem p. 31).

Finally, Laloux argues there is an emerging form of organization, which he calls evolutionary-Teal characterized by self-management, wholeness and evolutionary purpose.

I find Laloux’s conceptualization and idea of a developmental continuum – in the lives of organizations, in how individuals see organizations and in which organizational forms become dominant at various historical periods – valuable to understand the evolution of education systems and also valuable to understand the task of implementing educational change at scale.

Since the public education system is a product of the Enlightenment, along with Democracy and with the modern research university, one would hope that the most apt organizational form to describe it would be the achievement organization, with its respect for expertise, reliance on evidence, and healthy skepticism to arbitrary authority and dogma. If this is the minimum standard, leadership or management approaches reflecting magic, impulsive or conformist organizational forms would be pulling the institutions of education backwards, not helping them progress. To a great extent, the language of ‘delivery systems’ is an apt language for the Achievement organizational model, in particular in its reliance on evidence to support decision making and in making policies ‘visible’ in their operational strategy and carefully monitoring implementation while creating accountability for delivery and results. In this view, the education system is in fact an ‘implementation machine’, a good clockwork model created in a Newtonian world in which the laws of science help us understand events, predict them and control them. Relative to a conformist organization, characterized by the arbitrary use of power, where dogmas are unquestioned, this Achievement organization represents considerable progress, and since many education systems function as Conformist organizations, helping them transition to an Achievement stage seems like a worthy goal.

But the Achievement model can reflect a rather static when it comes to accepting social norms and institutions, more apt to reinforce the power and privileges of dominant groups than to challenge them, more fit perhaps to conserve social norms and institutions than to change them. The Punjab reform, as well as the reforms in Mexico, Kenya and Zimbabwe were top down reforms, they depended largely on strong support not just from education authorities, but from the President and the Prime Minister. Arguably the legitimacy of those leaders rests of the legitimacy of the process through which they reached their position and if the leaders are legitimate they should have the authority to ‘command’ an education delivery system to achieve the goals set for them, which to some extent reflect the goals of the electorate, the ‘mandate’ they have given their elected authorities. Other stakeholders however, parents, or teachers, or students themselves, may have various views on the legitimacy of political authorities to determine education goals or means. Even if they accept the legitimacy of the President or of the Prime Minister as political authorities, they may not confer them with the authority to represent the educational needs or interests of the students. There is an obvious conservative undertone to an Achievement organization, great to ‘get things done’, but perhaps not great to produce social change.

A view that sees all perspectives as deserving equal respect, as reflected in a Pluralistic view of organizations, can be at odds with the view of Achievement organization’s that those at the top should direct the organization. But isn’t this pluralistic view the perspective from which various liberation movements emerged, movements that advanced the rights of women, or people of color, of the poor, or those with different learning needs? If democracy is a living organism, it is because the notion of who ‘belongs’, of who has rights, is dynamic, contested, always in flux. A pluralistic perspective, or an evolutionary purpose view of organizations, creates the space that allows for that continuous evolution to take place.

How do these ideas about the nature of organizations relate to the reforms studied in this book? Each of these six reforms is based on an implicit view of the education system as an organization, the Achievement view is dominant. As such, each of these reforms serves as a self-fulfilling prophecy to further consolidate that education system into that organizational form. Can education reforms help systems ‘move forward’ towards more complex organizational forms? When I wrote ‘Educating Students to Improve the World’ the implicit view of educational institutions covered a narrow spectrum between Achievement or Pluralistic organizations, two view of organizations I had recognized in my earlier book ‘Informed Dialogue’, in which I had contrasted top down approaches to change with ‘learning organization’ approaches that honor the diversity of views of various stakeholders affected by education (Reimers & McGinn, 1997).

At present, I find Laloux’s theory about organizations considerably richer and more capacious as a way to enhance the Institutional perspective on educational change I included in ‘Educating Students to Improve the World’. I now think that the language of educational change as strengthening a ‘delivery system’ conjures up a particular mental model about the implementation process that is top down, command and control, perhaps a good fit to an Achievement organization in a rather stable and predictable world, but not nearly as adequate in a world in flux, where good ideas can come from anywhere, or for a Pluralistic or Evolutionary Purpose organization, where we must actively cultivate and honor dissent.

I can appreciate how an education reform that reinforces an Achievement model of organization represents progress in systems that need to be liberated from more primitive organizational forms, where capture of the public education system by private interests subordinates them to tribal forms of governance characteristic of Magic and Impulsive organization, or to the pre-Enlightenment conservative forces of Conformist organizations. Clearly the reforms in Mexico and Pakistan, in changing teacher appointments to make them more merit based, where inducing exactly that kind of transition from Conformist to Achievement organizational functioning. The reforms in Ontario, Pakistan and Mexico, in using data on student achievement to drive accountability and improvement where trying to replace arbitrary decision making, characteristic of conformist organizations with public evidence decision making characteristic of Achievement organizations. But it is unclear to me whether a reform that consists of building capacity by strengthening the capacity to do as told, helps in any way to evolve that system towards more pluralistic or evolutionary forms of organization, where multiple perspectives are valued, and where individuals can self-manage, be whole and achieve evolutionary purpose. The slow progress of the Ontario’s reform towards educating students for a breath of skills, in spite of the significant investments in building capacity an professionalism, are a cautionary tale on the power that reforms based on one implicit model of how organizations function to reinforce that particular model, and prevent evolution towards more complex forms of functioning.

To sum up, because implementation details in effect are what becomes of policy intent, and because implementation depends on our mental models about how change happens and about how education systems evolve, implementation matters greatly to thinking about the process of educational change. As those mental models become open and subject to scrutiny, they can inform more sophisticated theories of mind that can allow more effective communication, collaboration and collective learning, and perhaps support the development of education organization towards more complex pluralistic and evolutionary forms.

## Lesson 3. The Need for Operational Clarity

People cannot support, or carry out, that which they do not understand. The appeal of approaches to reform that focus on the core literacies rests not only on the fact that building and sustaining trust is easier if reformers commit to a few narrow goals and pursue them with laser-like focus, but also on the fact that because educators have focused on those goals for a relatively long time, there is widespread agreement about that these goals mean, about their importance, and about how to measure and even support them. Such focus on a few goals was the cornerstone of the British education reforms carried out during the administration of British Prime Minister Tony Blair, on which the reforms that McGuinty pursued in Ontario were inspired, and which inspired also the reforms of the Punjab. The similarities between the three reform are not accidental. Sir Michael Barber was one of the architects of the British ‘National Literacy and Numeracy Strategy’. He was also a key figure in the design and implementation of the Punjab reforms. In turn, one of the advisors to Ontario’s reforms, Michael Fullan, had studied the National Literacy and Numeracy Strategy, and was inspired by it in designing Ontario’s reforms, adding some important modifications in the form of more support for professional development.

To be sure, even as these three reforms prioritized the ‘basic’ goals of literacy and numeracy, there are some disagreements on what is meant by literacy – they form the basis of the well known ‘reading wars’ – or on the most appropriate approaches to advance them or measure them, but given that the scientific study of literacy spans many decades, those disagreements are minor compared to the state of consensus in domains such as self-efficacy, or perspective taking, or grit, or with respect to the vast set of constructs that are covered in the area of competencies for the twenty first century as discussed in the first chapter of this book. I recall once a conversation with the founder of the Partnership for Twenty First Century Skills, Ken Kay, in which he explained that as a result of working with various states in the United States advocating for broadening the goals of the education standards, he had realized that it was very difficult for most people to focus on a large number of goals, so he made the decision to narrow the focus of the Partnership’s advocacy, from a dozen or so outcomes to ‘Four Cs’ (Critical Thinking, Creativity, Collaboration and Communication). Perhaps this is also the reason UNESCO’s Commission on Education for the Twenty First Century, distilled 3 years of global consultations on the goals of education, and thousands of pages in background documents, to Four Pillars’ (learning to know, learning to do, learning to live together and learning to be). Michael Fullan also, whose recent writings reflect an expansion of his earlier focus on the basic literacies into a broader set of outcomes, focuses on six Cs: Character, Citizenship, Collaboration, Communication, Creativity and Critical Thinking (Fullan & Scott, 2014).

But it isn’t just the sheer number of competencies that a focus on ‘breadth of skills’ could require that makes some reformers leery of them, it is also the fact that there is less consensus on what those outcomes should mean, or on how to measure them. Clearly a focus on literacy and numeracy is a safer way to go if one is looking for a few clear goals, which don’t cause much controversy, and on which there is widespread agreement on how progress is to be measured.

The downside of excessive operational clarity on a few narrow goals is that it may unduly constrain the focus of improvement. At its worst, if a reform defines as its goals to improve student test scores, this could lead to a narrow instructional focus where teachers teach to the test or to other shortcuts to improvement, such as focusing on teaching to the children who are just below the cutoff test scores, such that the improvements which are measured are spurious. That is a frequent criticism of standards based reform as discussed in the first chapter. The challenge for reform is to be understood with sufficient depth that means are not conflated with end goals, so that no one reaches the absurd conclusion that learning is ‘that which the tests measure’. Especially if we view an education system from a pluralistic or evolutionary perspective, it is essential that all stakeholders can think with clarity about what are the goals that matter, and that they know the limits of any particular operational definition. This is hard to achieve, even in the context of reforms that invest heavily in teacher capacity and professionalism, as was the case in Ontario’s reform. Midway through this reform, which was initially narrowly focused on literacy and numeracy, the definition of those constructs was broadened to encompass broader skills. Even as more policy documents began to include references to a broader range of competences and deep learning, that expansion did not easily translate into implementation. As I will explain later in this chapter, the initial steps in a reform have a long lasting legacy that shapes much of the future reform trajectory.

## Lesson 4. Large Scale Reform Is a Journey: Coherence, Completeness and the Five Frames

If education reform is more about implementation than it is about design, it is obvious that changes to the day to day practices that constitute teaching and learning, for all students, at scale, is a demanding task that will require time. There are at least two reasons such time is necessary. The first, organizing and executing the activities that support such changes in educational practice takes time. The second, individuals, and organizations, need time to assimilate what they are learning in a way that translates into practice and eventually replaces ‘old ways’. This is the sense in which changing ‘the culture of education’, to use Jerome Brunner’s apt term, is a process of cultural change: possible, but slow. The dynamics of such process of educational change are aptly captured in the metaphor of ‘geological layers’ used by Tyack and Cuban in their study of education reform in the United States, in which they argue that federal mandates reach schools in the form of ‘geological layers’ that pile on top of layers created by previous mandates (Tyack & Cuban, 1997, 76).

If education reform is a journey it is necessary to (a) sequence activities, because not everything can be done at the same time, (b) for the journey to stay the course over time, and (c) to be able to course correct based on learning from experience. Sequencing, continuity and course correction require a reform that is complete and coherent. I now turn to explain how the five dimensional theory of educational change can support completeness and coherence in design, to then address the subjects of sequencing, staying the course and learning from experience.

Any reform is based on a set of assumptions about how the education system functions and how to change it. I have explained how those assumptions could be usefully categorized as five frames: cultural, psychological, professional, institutional and political (Reimers, 2020). A recognition of those frames could be useful in various ways to the design and the analysis of education reforms. First, it could help assess the internal coherence and completeness of the proposed reform in terms of the frame within which it was operating. Second, it could help develop a more robust reform by identifying blind spots in the proposed reforms as highlighted by the frames which had not been considered. Finally, it could help produce coherence in how various stakeholders understood the reform, and their particular role in it, as it provides a shared language and set of mental models that facilitated communication about the reform across the many people whose collaboration is essential in a large scale education reform. Coherence results when different stakeholders share an understanding of what a reform is attempting to do, how and what their own role in it is supposed to be. Laloux’s theory of the developmental stages in the history of organizations has profound implications for how we interpret coherence. For organizations which are in an Achievement developmental stage, or in a previous stage such as Conformist, lack of a shared understanding of what a reform is trying to do can be interpreted as lack of capacity of those tasked to implement it, or lack of power of those leading the implementation of the reform to persuade or coerce implementors to ‘do as they are told’. From this frame of mind, it makes sense that ‘training’ or using information to strengthen ‘delivery chains’ is a useful way to increase coherence. In other words, the root cause of lack of coherence is ignorance or lack of will to comply, and the response is capacity building or incentive and accountability structures to compel individuals into compliance.

However, from the more complex worldviews of organizations represented by pluralistic or evolutionary purpose organizations, following Laloux’s taxonomy, lack of coherence could be more than the mere expression of ignorance or unwillingness to comply, as rightly articulated by Lipsky in his study of street level bureaucrats. Those organizational forms require valuing different perspectives of those in the organization, and continuous learning together, which in turn require a theory of mind, the capacity to understand the beliefs, intentions, knowledge and worldviews of others. Because different individuals may operate from various of the five frames (cultural, psychological, professional, institutional and political) integrated into my theory of educational change, such theory provides the means to develop a more complex theory of mind for each member of the organization, in understanding and appreciating the perspective of others, and consequently a capacious tool to facilitate communication, collaboration, and join learning. Such complex theory of mind is essential for teams to be able to recognize the shortcomings of any theory of action underlying a reform, and to identify ways to evolve together towards more a more capacious theory of action.

## Lesson 5. Sequencing, Pacing and the Importance of First Steps

Deciding which particular components of a reform to address, and at what speed, requires reconciling the ambition of the reform goals and activities, with the existing capacity and resources. It may also require attention to the developmental stage of the organization. Some activities logically need to precede others, for instance, it is necessary to have clear standards before student knowledge and skills can be meaningfully assessed. From that perspective, it was contradictory that Mexico’s reform developed a model of teacher assessment before the curriculum model that guided the reform was available, just the opposite of the more logical sequence Singapore followed in creating a framework for twenty-first century teacher education. Similarly, some organizational or individual capacity needs to be developed before certain functions can be carried out, for example, some coordinating mechanism of the functions of teacher selection, initial teacher education and in-service education, needs to be established in order to intentionally strengthen the continuum of teacher professional development. Without such institutional forms, Singapore could not attempt to develop a twenty-first century model of teacher, for example.

Is it necessary for education systems to stage reforms so that they first focus on creating the capacity to deliver simpler goals, before they can take on more ambitious goals? The reform in Punjab, for instance, focused on fairly narrow access, literacy and numeracy goals, as did the Ontario reforms which focused on literacy, numeracy and graduation rates. In neither case did the reform evolve towards addressing broader goals than those that were initially established. In contrast, the reforms in Singapore, Mexico, Kenya and Zimbabwe are all focused on broader education goals, even though it is unclear that any of them, except Singapore, are making progress towards changing the culture of education towards a broader set of outcomes, although in fairness Kenya and Zimbabwe’s reforms are in too early stages of implementation to be able to expect impact on teacher practices or student learning outcomes.

From an organizational developmental perspective, are Mexico, Kenya and Zimbabwe’s reforms attempting to pull above their weight in pursuing reforms to develop twenty-first century competencies? Would they be best served pursuing narrower goals, and using increased capacity gained from achieving those goals as a way to develop implementation capacity, to only subsequently pursue more ambitious goals? That seems to be the underlying assumption of the Punjab reforms. While the idea of taking small steps first, focusing on narrower goals, has intuitive appeal, Ontario’s reform seems to disconfirm the notion that systems naturally transition to more complex and ambitious goals as they succeed in improving narrower goals. Even though the reform was quite successful in achieving large scale improvement in literacy, numeracy and graduation rates, and was sustained for a fairly long period of time, it has not yet transitioned to adopting a broader set of goals or implementing the necessary changes so that the culture of schools pursues them, even though one of the architects of the reforms (Fullan), who remains an influential thought leader in the field of system level change for educators of all political persuasions in the province and in the world, has increasingly written about the importance of pursuing such more ambitious goals.

Kenya’s experience with the pilot of the new curriculum revealed that the needs for professional development had been underestimated and that the initially planned pace of the reform was too ambitious for the capacity of the system. The aim to change the curriculum, requiring more personalized instruction, while also changing the structure of the cycles of education, with automatic transition from primary to secondary schools, in a short timeframe, seems to have exceeded the capacity of the system with the logistical requirements of implementing such changes. For instance, the combined effect of new pedagogies, relying on ongoing formative assessment of student progress with much larger secondary classes created by the elimination of the exams created demands that are difficult to meet by the current teacher force.

In sequencing the implementation of a reform three critical considerations should include the ambitions of their goals, the existing level of implementation capacity, and the stage of development of the education system. Laloux characterizes the development of organizations as an evolutionary, staged, process, where certain stages in organizational development preceed higher levels. It may not be realistic to expect education systems functioning as proto-Achievement organizations, for example functioning within the rigid hierarchical stratification of conformist organizations, to carry out activities that require a pluralistic organizational mindset, without first helping them evolve into an achievement organization. In Laloux’s description of a conformist organization I recognize the practices I have observed in many ministries of education, in fact Laloux’s description is close to the way my colleague Noel McGinn and I characterized the internal structure of education systems in the book Informed Dialogue (Reimers & McGinn, 1997). What a huge leap forward it now seems,with hindsight, to expect the institutions operating out of that conformist worldview to transition into a professional culture of self-management, or even into the modes of democratic participatory decision making I advocated in the same book, or that are implicit in the ideas of schools as learning organizations or in the concept of collaborative professionalism advanced by Andy Hargreaves and his colleagues (Hargreaves, Shirley, Wangia, Bacon, & D’Angelo, 2018).

In a conformist organization: “Planning and execution are strictly separated: the thinking happens at the top, the doing at the bottom. Decisions made at the top get handed down through successive layers of management …. A whole catalog of rules is set up. Some among the staff are put in charge of ensuring compliance and handing out disciplinary measures and punishments for those found wanting … The underlying worldview is that workers are mostly lazy, dishonest, and in need of direction. They must be supervised and told what is expected of them. Participatory management seems foolish from a Comformist-Amber perspective; management must rely on command and control to achieve results. Jobs at the frontlines are narrow and routine-based. Innovation, critical thinking, and self-expression are not asked for (and often discouraged). Information is shared on an as-needed basis. People are effectively interchangeable resources; individual talent is neither discerned nor developed” (Laloux, 2014, 21–22).

While I endorse holding the aspiration that all education systems should become, in time, pluralistic or evolutionary purpose organizations, I recognize it may take some intentional sequencing to help some systems get there. Education reforms are the conduits to support that transition. I do not know, at this point, whether it is possible to plan for systems to ‘leapfrog’ stages in Laloux’s sequence, but I do think there has to be alignment between the tasks and expected functioning in any particular stage of education reform and the organizational arrangements and practices that characterize how the education system functions. Those can change, just as ‘the culture of education’ can change, but slowly, with adequate support and skillful design.

From this perspective, a key difference between the reforms in Mexico, Punjab, Ontario and Singapore, are not just that Punjab and Ontario pursued rather narrow curricular goals, whereas Mexico and Singapore pursued more ambitious goals. But another fundamental distinction is that Mexico and Punjab’s education systems were both in a proto-Achievement stage, whereas Ontario and Singapore where in an Achievement stage proper. Punjab’s reform made as much sense as an instrument to help that organization transition to an Achievement stage as it did as an avenue to pursue improvements in access and literacy. Mexico’s reform was also instrumental to help the system evolve into an Achievement stage, while pursuing a much more ambitious set of educational goals. The sequence followed by the Mexican reform made more sense as a path to help that organizational transition, than as a sequence to achieve a twenty-first century curriculum reform.

In contrast, Ontario and Singapore, both Achievement organizations, pursued goals of different level of ambition, narrower in Ontario than in Singapore. It is not evident that either of these reforms attempted to help those systems transition into a Pluralistic or Evolutionary Purpose stage, instead the reforms helped to consolidate those systems into an Achievement stage. With hindsight, Ontario might have more readily transitioned to teaching twenty first century skills following the intentional staged process followed by Singapore, rather than expecting that the successful implementation of a strategy focused on narrow goals would eventually lead the system there. Conversely, Mexico might have benefited from attempting a transition to an Achievement organization focusing on a narrower set of goals, as did Ontario, rather than attempting similar goals to Singapore’s with an organization at a very different level of functioning.

On the other hand, Ontario, Punjab and Singapore benefited from sustained periods of educational continuity which eluded Mexico, and perhaps also Kenya and Zimbabwe, for reasons that are somewhat extrinsical to the particulars of the education reform, and contingent on national politics and economic developments. To some extent the role played by those external political circumstances, a change in government, government continuity, are not within the control of education reformers. We might think of them as just pure luck, good or bad. But perhaps there are steps that could increase the probability that a reform will be long lived, other than counting on good luck.

If there is something education reformers can do to secure the necessary continuity of a reform it is attending to the ‘narrative’ of reform, as it will inform how stakeholders at various levels of the system make meaning of it. In building a reform’s narrative first steps are critical. They set the initial conditions of the reform in a way that will signal for many what the reform is about, and that can open possibilities for subsequent steps, when they build capacities or create essential units, as much as they can close them. The Mexican education reform provides a great example of the necessity of sequencing and of the importance of first steps. The reformers realized that before expecting teachers to be able to implement an ambitious curriculum reform, which required among other conditions high levels of professionalism and more autonomy at the school level, the norms that governed who could become a teacher should be aligned with a professional view. To this end, they eliminated the control of the teacher union over the process of teacher appointments, in favor of giving the state the authority to assess knowledge and skill of candidates to enter and remain in the profession. In terms of the evolutionary sequence described by Laloux this effort attempted to replace organizational practices appropriate to impulsive organizations, characterized by sheer and continuous exercise of power in interpersonal relationships to sustain the position of the chief, with practices aligned with conformist organizations, or even of proto-achievement organizations. The direction of the proposed change was sensible, at least using Laloux’s theory, even though there were incongruencies with what further elements of the reform expected. For instance, the curriculum reform which was eventually approved, expected a level of teacher professionalism, and school autonomy, that would have required mindsets and practices typical of Achievement and Pluralist organizations.

But the challenge with beginning the reform replacing the process to appoint and promote teachers with one based on merit is that it allowed the opposition of the reform to see it as a ‘punitive’ reform, as a reform that was about an attempt on the part of the State to grab power over the teaching profession. That narrative allowed those opposing the reform to build sufficient support to slow the reform down, and eventually to cause the leading opposition presidential candidate to commit to undoing the reform. Even though this reform could have just as easily been framed as a reform to promote teacher professionalism, to increase school autonomy, to recognize a plurality of legitimate views about the goals of education, which were indeed goals reflected in elements of the reform, that narrative never crystalized because the elements that most clearly reflected them came later, with hindsight too late. Once the ‘New Educational Model’ was announced, the element of the reform most explicitly focused on curricular goals, several consultations were conducted. While there are clear parallels with the Ontario reform in how Mexico created opportunities for teacher participation and support, the key difference between both reforms is the moment at which those opportunities for participation were deployed. In Ontario, they happened very early, marking a start contrast with the conflicted relationship between teachers and government which had characterized the administration preceding the reform and therefore shaping the narrative of the reform from the outset. As a result the reform was perceived as ‘educational’ and as ‘inclusive’ even though it was as political as any of the other reforms examined in this book. In contrast, in Mexico, they happened late, 3 years into the reform, once a narrative about the reform had developed and opposition had formed and organized against it. While both of these reforms could claim that they sought teacher participation, the devil, to quote Fullan and Gallagher, is in the details of how *and when* this was done (Fullan & Gallagher, 2020).

## Lesson 6. Staying the Course

Reform takes time, the most elusive of all resources. Time is needed to carry out all the activities that have been carefully sequenced to allow a reform to reach its goals. Time is needed for the policy mandates of a reform to eventually crystalize into a new pedagogical ‘core’ that effectively shapes the new ‘culture of education’. But time is by definition elusive in a world that is volatile and uncertain. The COVID-19 Pandemic likely derailed the best laid plans of many educational institutions, forcing them instead to deal with a world with new demands and different means to deliver education.

Political discontinuities are the main threat to the stability that reforms need to consolidate. When a president changes, the minister of education is likely to change. When the minister changes, senior leadership teams can change. With those changes, the balance of power that supported the reform may change. There are clear benefits from long policy cycles, and big costs to rapid interruptions of reforms, before those have had a change to bear fruit. What accounts for continuity in education policy cycles? Is such continuity something that can only be recognized after the fact, a product of good luck perhaps, or is it something that can be planned a priory, that can be cultivated and sustained over time?

One key element influencing implementation continuity are national politics and their relationship to education. Political support from high levels of government is not only helpful, but frequently essential. The magnitude of the changes required to put in place and sustain an education reform to augment the ambitions of the curriculum are such that they require substantial financial resources and political capital. Singapore’s long cycles of reform have benefited from considerable support from the Prime Minister. In Ontario the fact that Premier McGuinty made education a priority of his administration since his arrival in office in 2003, and political continuity and stability for a decade, were very valuable to sustain the priority of the reform, the continuity of key teams and a steady influx of resources. The strong political support for reform in Singapore or Ontario never became a liability, even when governments or education administrations changed. In contrast, the Mexican reform is a clear example of how the same political support that benefited the initiation of a reform, heavily identifying it with a particular administration, became a liability when a presidential transition to a candidate from an opposition party took place. The Mexican reform began as one of the key structural reforms that an incoming administration launched to restructure the State. It was perceived as central to the strategy of the administration, and benefited from considerable support from the administration for that reason. At the same time, such high level of support identified the reform with the administration, and made it an obvious potential target for groups opposing the President. When an opposition candidate launched his campaign against the incumbent party, the education reform was an easy political target, and opposing it allowed that candidate to obtain the support of groups opposed to the education reform. Dismantling the education reform became a campaign promise, even though the interests motivating the opposition were more about dismantling any legacy of the previous administration than specific interests around the education goals or strategies of the education reform per se. A reform less visibly identified with a Presidential administration would have made a less likely target in an electoral campaign. When the opposition candidate was elected President he had to fulfill his electoral promise.

Political discontinuity also ultimately interrupted Ontario’s reform, but did so after a long period of implementation, which allowed enough of the culture of education to have changed for some of that change to continue under the leadership of schools and local organizations. In addition to the good fortune from that reform resulting from the continuity of a party in power, Ontario’s reformers explicitly framed the reform as an educational reform from day one. They also intentionally sought to gain trust and support from parents and teachers early on. Mexico’s reform was more top down, and insufficiently focused at the outset on curriculum and pedagogy, which was paradoxical for a twenty-first century education reform. The almost exclusive focus on institutional changes, important as those were both as foundations to the pedagogical reforms and as avenues to help the education system develop into more advanced organizational forms, allowed a narrative to develop about the reform from which it would never recover. That fact, and the limited implementation of the reform by the time a new government came into office made the reform very vulnerable to severe interruption.

The reform in Punjab, also benefited from a relatively long period of political stability in the province, and from consistent high level support from the Prime Minister. That kind of support and continuity were essential to the significant changes this reform enacted, changing rules to appointing teachers, for example, towards greater meritocracy, or increasing the number of local school supervisors who would work as coaches and pedagogical advisors in schools.

As an example of the benefit of long policy cycles to the implementation of a reform Singapore stands in a class of its own. It is clear that to some extent such continuity has resulted from the very long period in power of the ruling party, and from the very high political priority that education has received from the nation’s leaders. Here too initial steps are very consequential. Lee Kwang Yew, Singapore’s first Prime Minister, built two very powerful narratives in the early history of the nation that to this day provide impetus to education. One was that Singapore’s future depended on the cultivation of the talent of the population, and that education was a cornerstone of strategic importance to shaping that future. The second was a certain modesty and awareness of the limited resources available in the early years of the nation’s history, which cultivated an approach to reform consisting on building on what had been done previously, rather than replacing previous reforms. The narrative that the country’s education system has evolved through four distinct phases, intentionally pursued, and that this has produced great results, sustains a view of reform as incremental continuous improvement, rather than one fell swoop change. In addition, the relatively small size of the nation state and of the education community, and the close ties between many who work in various roles in education, sustains a close knit profession where there are strong incentives to be respectful of the institutions, policies, programs and people that are in place and cautious in attempts to make too many changes at once. As a result, Singapore has, arguably, the strongest institutional education culture of all six countries examined here and this, in itself, is a driver of continuity and long policy cycles.

While support from high level political leaders, such as the support the reforms in Ontario and the Punjab received from their respective Prime Ministers, or the support the reforms in Kenya, Mexico and Zimbabwe received from their respective Presidents, are undoubtedly helpful, ultimately earning the support of students, parents and teachers may be more important to the long term continuity of the reform. The Ontario reform explicitly sought to earn the trust of society and of teachers, this may account for the significant continuity of the reform over a decade. It is difficult for elected leaders to undermine reforms which are widely valued by the voters. The Mexican reform, in contrast, does not appear to have invested as much in earning such support, while its detractors actively worked to undermine such trust, building a narrative that portrayed the reform as ‘top down’ and ‘punitive’. The Punjab reform has lost support as a result of a change of political administration in the province, and because it never sought to gain support from teachers and parents, as Ontario’s reform did, it is more fragile. Zimbabwe appears to have more intentionally sought support from the population by opening up consultations about the new curriculum than Kenya, but it is unclear how the larger political instability will influence such support and the durability of the reform.

## Lesson 7. Learning from Experience to Build System Level Capacity

As explained earlier in this chapter, the theory of action of any reform is series of hypotheses about how it is that certain activities will produce certain immediate results and in turn contribute to larger educational goals. Good implementation of educational change is continuous course correction, adjustments based on what is learned as a result of attempting what the reform proposes. If programs of professional development are offered, will teachers participate? Will they learn from them? Will the programs contribute to changing their practice? If they change their pedagogy, will students develop new competencies as a result?

The reason methodologies such as the logical framework approach are useful is because making those hypotheses visible and public helps develop a shared understanding of what it is that a policy is trying to do and how. But the logical framework is also a management tool, helping to focus attention on the key activities that translate a policy or program into an implemented reality. Just as important, the logical framework is a tool to support evaluation, and continuous program improvement.

The pilot of the reform which Kenya implemented in 470 schools was a deliberate effort to test the new curriculum, teacher preparation, new assessment, in a diversity of contexts and levels. The use of continuous assessment in the pilot served to provide regular feedback to help develop shared understanding of what the curriculum reform would look like in practice and to make necessary adjustments as a result of what was learned.

Learning from the process of implementing the reform is essential not just to adjust the reform so it better achieves its goals but also to achieve coherence among the many stakeholders that must collaborate to make change happen. How we think about who precisely it is that must learn what depends on our mental models of organizations. In a Conformist organization, using Laloux’s language, which supposes a clear differentiation between those who make policies and those who do as they are told in implementing them, those who make policy need to learn whether the hypotheses on which the reform is based are correct, and whether they are been implemented as intended, while those who implement it need to learn what is expected of them, and need to develop the skills necessary to do as they are told. In contrast, in an Achievement, Pluralistic or Evolutionary Purpose organization, people need to learn together whether the theory of action holds up. In those organizations good ideas can come from anywhere, and the insights of teachers, or of students, can be as valuable as those of a Minister. In addition, in Pluralistic organizations, people need to learn together whether the goals the policy is pursuing make sense, or whether they are sufficient or should be expanded. In an Evolutionary Purpose organization, they may also need to learn together whether the reform is helping advance the education system to higher levels of evolutionary purpose, or holding it back.

An example of a ‘learning organization’ perspective, reflecting a pluralistic or an evolutionary purpose view of organizations is provided by Michael Fullan and Mary Jean Gallagher in their recent book ‘The Devil is in the Details’. These authors explain that coherence requires that people ‘understand the system’ in order to collaborate towards good system change. In their view good system change is the result of coherent work of people working at three interrelated levels of the system (the macro, the middle and the local). As people become aware of the worldviews of those in their same level, as well as of the worldviews of those in the other two levels, they can understand the system in which they work and come to understand the system dynamics of educational change (Fullan & Gallagher, 2020, 32–33).

The Ontario and Punjab reforms, which included accountability and data as instruments of school improvement, explicitly built into the theory of action of the reform feedback loops for improvement. In Ontario, for example, the assessment results were used by teachers to identify the extent to which each of the students was meeting curriculum expectations, and those data were used by principals to develop school improvement plans. Schools received data on the performance of their students, compared with the performance of students in other schools with similar demographics. The results were also used by school improvement boards and by the ministry to launch support initiatives. In this way, the same information, shared and used for different purposes across the three levels of the education system, helped shape a shared understanding of what the challenges were with respect to achieving the reform objectives.

In Punjab, the most significant activity to strengthen management capacity consisted of creating a monitoring and reporting system of all key actions involved in the implementation of the reform. Those reports were frequently discussed by teams at all levels of the implementation ‘delivery chain’ to facilitate course correction but also learning. The parallel with the approach followed in Ontario is not accidental, as that reform too, just like the reform in the Punjab, borrowed this emphasis on data utilization to strengthen delivery from the National Literacy and Numeracy Strategy in the United Kingdom.

In contrast, implementation of the Mexican education reform was hindered because states, which have substantial authority in education governance, had an uneven commitment to the reform. The top down nature of the reform, with most of the impetus coming from the Federal government, and with limited opportunities for input from the local and state level, prevented the development of shared learning of stakeholders across these various levels, resulting from access to similar information or from collaboration in efforts of improvement as was done in Ontario or in Punjab. The result, predictable, was limited coherence and limited buy-in and support from local and state levels.

## Coda

The year 2020 was a pivotal moment in the history of humanity. Not only did the COVID-19 Pandemic extract a heavy toll on many lives and on societies, it disrupted many of the existing processes and social institutions that defined life as we knew it. The effects of that disruption are likely to be felt for a long time to come, even after the Pandemic is over. To a great extent, the educational costs of the Pandemic caused significant losses for individuals and nations. Some may never fully recover from those losses. These losses are all the more reason to reassert the importance of education for the future of humanity. This new awareness about the importance of education and about the need for reinvention present a challenging conundrum. The urgency to reinvent education will happen in a world heavily burdened by the results of the Pandemic, not least among those burdens will be the financial austerity that is likely to follow. We do not yet know how the Pandemic will accelerate pre-existing challenges and create new ones, but the study of past Pandemics teaches us that the challenges they create can have profound consequences for society.

A global pandemic is one of the most humbling experiences we could experience. In some ways a great equalizer, for a highly contagious virus knows no borders and no differences among classes of people, even though different forms of privilege influence the likelihood of contracting the virus and the fatality of losing one’s life to the infection. Even so, that so many people around the world saw their freedoms constrained by this Pandemic provided a humbling reminder to all of our shared humanity, and an opportunity for deep empathy with others, of what it is to live without whatever privileges we may have grown accustomed to. It may have also provided time for some for deep thinking and reflection, for more attention to questions of purpose, of deep meaning, including questions about the purpose of education, in which societies invest so many resources and effort and in which we place so many hopes. Perhaps this global calamity will help us grasp the deep meaning in the powerful idea Terence put forth twenty centuries ago: *‘to be human is to live so nothing human is foreign to us’*. Perhaps this crisis will remind us of the importance of solidarity, of the power of collaboration, of the power of reason, of the importance of science, or the power of good leadership, and above all of the need to make ethical choices.

In 1347 a ship brought the bubonic plague to Italy. In a year 25 million people had died. Over the ensuing decades, some of the social and economic disruptions caused by the Pandemic stimulated many questions about the pre-existing social order, along with an unprecedented social and economic mobility and many questions about the absolute authority of rulers, represented in the rise of the influence of the Medici family in Florence, where they would become patrons of a massive experiment in intellectual cross-fertilization that would in time bring about the Italian Renaissance.

In contrast, the 1918 Pandemic in Germany depressed municipal spending in ways which created challenges for the emerging Weimar Republic, and which radicalized many of those who felt excluded as their lives were negatively impacted by the economic impact of the Pandemic. Many of them would in time develop extremely intolerant views which contributed to the rise of intolerance, the development of the Nazi party and the breakdown of democracy (Blickle, 2020).

Whether our way out of this crisis goes one way or another, a Renaissance or a breakdown of democracy and social institutions, depends on how people make sense of the painful and confusing disruptions the Pandemic has brought about. Education can play a critical role helping people make sense of this time, and shaping the ethical choices that will define how we live after this crisis. Education will be critical so that at the end of this crisis there is a Renaissance, and not a return to the dark ages.
